# The Swedish HealthPhys Study: Study Description and Prevalence of Clinical Burnout and Major Depression among Physicians

**DOI:** 10.1177/24705470221083866

**Published:** 2022-04-04

**Authors:** Emma Hagqvist, Kerstin Ekberg, Ulrik Lidwall, Anna Nyberg, Bodil J. Landstad, Alexander Wilczek, Fredrik Bååthe, Malin Sjöström

**Affiliations:** 1 Unit of Occupational Medicine, Institute of environmental medicine, 27106 Karolinska Institutet, Stockholm Sweden; 2 Department of Health, Medicine and Caring Sciences, 4566 Linköping University, Sweden; 3 Division of Insurance Medicine, Department of Clinical Neuroscience, 27106 Karolinska Institutet, Stockholm, Sweden; 4 Official statistics Unit, Department for Analysis, Swedish Social Insurance Agency, Stockholm, Sweden; 5 Department of Public Health and Caring Sciences, 8097 Uppsala University, Uppsala Sweden; 6 Department of Health Sciences, 90835 Mid Sweden University, Sweden; 7 Unit of Research, Education and Development, Östersund Hospital, Östersund, Sweden; 8 Department of Clinical Sciences, Danderyd Hospital, 27106 Karolinska Institutet, Stockholm Sweden; 9 87437 Institute for Studies of the Medical Profession LEFO - Legeforskningsinstituttet, Oslo, Norway; 10 Institute of Stress Medicine at Region VGR, Gothenburg, Sweden; 11 Institute of Health and Care Sciences, Sahlgrenska Academy, Gothenburg University, Gothenburg, Sweden; 12 Department of Public Health and Clinical Medicine, 59588 Umeå University, Umeå, Sweden

**Keywords:** burnout, COVID-19, depression, physicians, Sweden

## Abstract

**Objectives:**

The study purpose was to describe the Swedish HealthPhys cohort. Using data from the HealthPhys study, we aimed to describe the prevalence of clinical burnout and major depression in a representative sample of Swedish physicians across gender, age, worksite, hierarchical position, and speciality in spring of 2021, during the third wave of the Covid-19 pandemic.

**Method:**

The HealthPhys questionnaire was sent to a representative sample of practising physicians (n = 6699) in Sweden in February to May of 2021 with a 41.3% response rate. The questionnaire included validated instruments measuring psychosocial work environment and health including measurements for major depression and clinical burnout.

**Results:**

Data from the HealthPhys study showed that among practising physicians in Sweden the prevalence of major depression was 4.8% and clinical burnout was 4.7%. However, the variations across sub-groups of physicians regarding major depression ranged from 0% to 10.1%. For clinical burnout estimates ranged from 1.3% to 14.5%. Emergency physicians had the highest levels of clinical burnout while they had 0% prevalence of major depression. Prevalence of exhaustion was high across all groups of physicians with physicians working in emergency departments, at the highest (28.6%) and anaesthesiologist at the lowest (5.6%). Junior physicians had high levels across all measurements.

**Conclusions:**

In conclusion, the first data collection from the HealthPhys study showed that the prevalence of major depression and clinical burnout varies across genders, age, hierarchical position, worksite, and specialty. Moreover, many practising physicians in Sweden experienced exhaustion and were at high risk of burnout.

## Introduction

A recent systematic review including 182 articles across 45 countries show high prevalence of common mental problems, eg, burnout and depression among physicians.^1^ In fact, the prevalence of mental problems and suicide are higher among physicians than in the general population.^2^ The systematic review^1^ concludes that the prevalence of burnout and depression varies across countries and that it is important to study contextual differences. To the best of our knowledge, no study has explored the prevalence of clinical burnout and major depression among physicians in Sweden nor potential contributors to clinical burnout and major depression in physicians working life.

To gain better knowledge about the Swedish physicians’ health and working conditions the project Healthy Physicians Sweden, the Swedish HealthPhys study was set up with the purpose to identify the prevalence of clinical burnout and major depression among Swedish physicians and explore factors contributing to burnout and depression. In this paper, we describe the HealthPhys study design and give a brief overview of the cohort (presented in “The HealthPhys Study”).

Based on data from the Swedish HealthPhys study, we aim to identify the prevalence of clinical burnout and major depression among Swedish physicians across gender, age, worksite, hierarchical position, and specialty (presented in “What Is the Prevalence of Clinical Burnout and Major Depression among Swedish Physicians?”). In the HealthPhys study, burnout is defined as “a work-related state of exhaustion that occurs among employees, which is characterised by extreme tiredness, reduced ability to regulate cognitive and emotional processes, and mental distancing”.^3^ Major depression is defined in the International Classification of Diseases (ICD) 10 and Diagnostic and Statistical Manual of Mental Disorders (DSM) IV, as a list of symptoms that patients could have. It is not necessary that patients have all of these symptoms for a burnout diagnosis.^4^ In a recent review by Rotenstein et al.,^1^ results show that estimates of overall burnout among physicians ranged from 0% to 80.5% across studies. Authors argue that the wide range mark the heterogeneity in burnout ascertainment methods, definitions, and outcomes, as well as statistical heterogeneity.^1^ Another reason could be geographical differences and variations across sub-groups of physicians, justifying that comparisons across groups of physicians are important.

Since the start of the Covid-19 pandemic, the high work demands have contributed to a further deterioration of the mental health among healthcare staff.^5–7^ During the pandemic, physicians worldwide have experienced more burnout, anxiety, insomnia, and depression than before.^8^ This study present the prevalence of clinical burnout and major depression among Swedish physicians during spring of 2021, that is during the third wave of Covid-19.

## The HealthPhys Study

### The HealthPhys Study Design

The HealthPhys questionnaire was developed and distributed to a representative sample of physicians in Sweden. The data collection took place February through May of 2021. Statistics Sweden drew the sample from the Swedish Occupational Register based on the Swedish version of ISCO-08, ie, the Swedish Standard Classification of Occupations (SSYK2012), and the Swedish version of NACE rev.2, ie, the Swedish Standard Industrial Classification (SNI 2007). Statistics Sweden was responsible for the distribution of the questionnaire and collection of data. They also made an analysis of missing cases and calculated weights. The study was approved by the Swedish Ethical Review Authority (2020-06613).

#### Sample and procedure

The population in focus were professionally active physicians in Sweden (N = 34 604). A sample was drawn from The Swedish Occupational Register year 2018. A stratified random sampling method based on 12 strata were used. For a geographical stratification, the population was stratified based on 6 administrative healthcare regions. Furthermore, worksite was applied to two strata either primary care facilities or hospitals. Based on 12 strata and a 50% response rate, a power calculation suggested a sample of 7200 physicians.

In February of 2021 an invitation letter was posted to the home address of the selected physicians with information of the project and on how they could participate by answering the questionnaire on-line. With two to three weeks apart, reminder letters were posted to those who had not already answered the questionnaire. With the second reminder a paper version of the questionnaire was included. Most (80.5%) answered the questionnaire on-line.

#### Questionnaire development

The questionnaire was developed based on international literature on physicians’ health and working conditions, along with research and theories in the field of working life and health as well as experiences from the research consortium. The questionnaire was piloted by a total of 12 physicians with varying specialties and hierarchical position. The pilot was conducted in three steps. First, it was read by one physician making comments to language and content. Thereafter adequate changes were made, and the questionnaire was distributed to two other physicians. This process was repeated until satisfaction at which point it was distributed to five physicians. Thereafter a statistician at Statistics Sweden made an additional overview of the measurements in the questionnaire.

In total, the questionnaire contained 79 numbered questions and 269 items. It consisted of four sections:

Section A, medical and occupational background: place of education, years as a physician, work hours, on-call work, workplace, research and educational activity, hierarchical position, and specialty (based on the specialties defined by the Swedish National Board of Health and Welfare).

Section B, work environment: demands (emotional, cognitive, and quantitative), work time control, and support from Copenhagen Psychosocial Questionnaire,^9^ organisational justice,^10^ leadership climate,^11^ illegitimate tasks,^12^ effort reward imbalance (ERI),^13^ psychosocial safety climate (PSC-4),^14^ moral stress,^15^ harassment, and discriminations (developed from the Swedish Work Environment Survey), workplace incivility scale (WIS),^16^ and work-life conflict.^17^

In addition, due to the Covid-19 pandemic, additional questions were included that covered physical and psychosocial occupational risks directly associated with work during the pandemic. The questions regarding the Covid-19 pandemic were developed from interviews carried out with 40 physicians about their experiences of working during the first wave of the pandemic. The interviews were conducted by the research group.

Section C, health: burnout assessment tool (BAT),^3,18^ the symptom checklist-core depression (SCL-CD_6_),^19^ general health,^9^ self-rated sickness absence and presenteeism, use of psychotropic medicine and alcohol and drug use.

Section D, demography: family constellation. To reduce number of questions Statistics Sweden added demographical variables (gender, age, country of birth and municipality of living) from the Swedish population register to the data file.

Data from the questionnaire will be linked to sick leave data from registers held by the Swedish Social Insurance Agency.

### The HealthPhys Cohort

**Figure 1.**
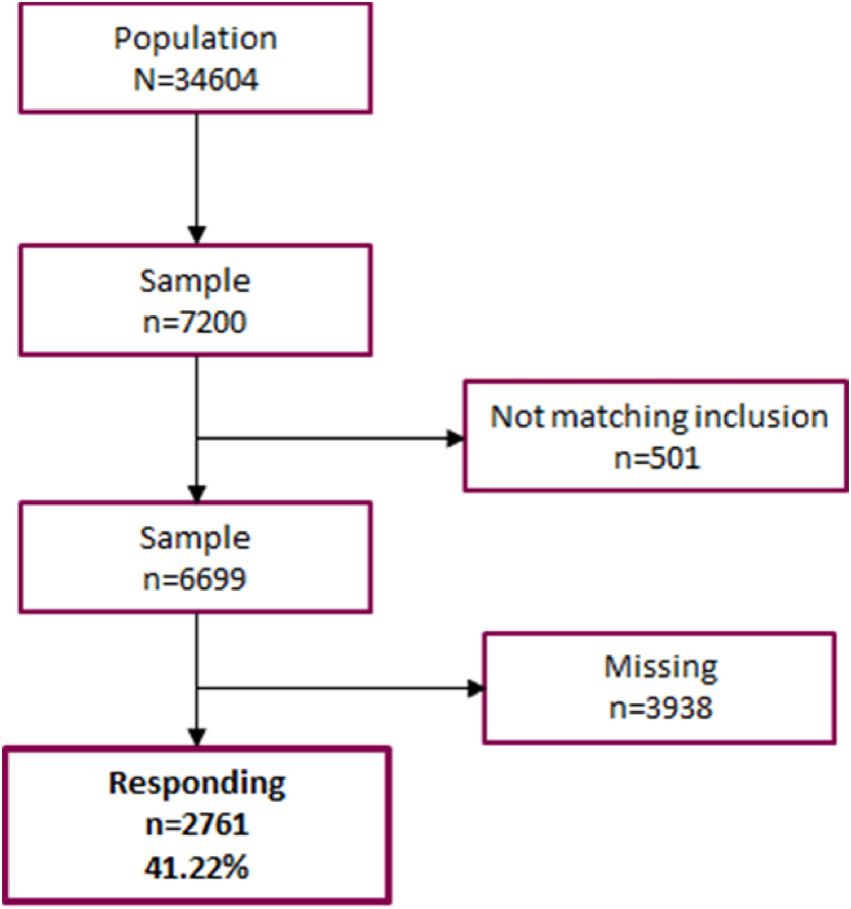
Population and sampling.

A total of 7200 physicians received an invitation to participate in the HealthPhys study (Figure 1). 501 answered that they did not match the inclusion criteria ie, had not been working as a physician in Sweden at any point during the previous 12 months, and were therefore removed from the sample leaving 6699 respondents. Of these, a total of 2761 respondents answered the questionnaire (41.2%). Response rate in the 12 strata ranged from 34.1% to 46.9%. Among those not responding (3938) did 25 decline to participate, 55 were not able to answer due to eg, illness and 37 were not reachable ie, invitation letter was returned to sender and 3821 did not respond. The sampling procedure is visualised in Figure 1.

To minimise the nonresponse error in analysis, weights were calculated. Calibrating weights was calculated according to Lundström and Särndal^20^ and enabled us to draw conclusion of the population of physicians in Sweden within a 1.9 per centage points margin. The description of the cohort is based on figures without weights applied.

#### Demographic description of the HealthPhys cohort

The HealthPhys cohort contain of 44.8% male and 55.2% female physicians. Mean age of the cohort was 47.5 years (St. deviation 12.1 years) ranging from 27 to 77 years (Mode was 37 years). Most physicians in the sample were born in Sweden (78.6%) followed by other European countries (15.8%) and non-European countries (5.6%).

Looking at family constellation, most physicians in the cohort had a partner (89.7%) and almost all of those with a partner were living with him or her (94.0%). About six in ten (60.7%) had children living at home and most of those had two children living at home (46.5%).

#### Occupational characteristics of the HealthPhys cohort

In the HealthPhys cohort, 40.6% of physicians in the sample were working in primary care facilities while 59.4% worked in hospitals (share of primary care-based physicians applying weights was 23.6% corresponding to national figures). Among primary care-based physicians, 43.7% were men and the corresponding figure in hospital-based physicians were 48.4%. Among primary care-based physicians, 26.6% worked in private facilities while the equivalent number for hospital-based physicians was 4.4%. In the HealthPhys cohort, 49.8% had more than 15 years of experience working as a physician. Almost 30% of the responding physicians mix clinical work with research or teaching activities in their employment.

Medical students in Sweden need to do an internship for 18 to 21 months before they can apply for a licence to practise medicine in Sweden. Only a small number, 33 of the respondents, stated that they were not yet licenced physicians and 42 (2.7%) were between general training and specialist training. Among those with a licence, 27.0% were resident physicians, 39.3% specialists or attending physicians and 29.4% were consultants which is the most senior hierarchical position. Table 1 show the number of physicians across hierarchical position as well as the share of women in each hierarchical position. There were more female physicians among junior, resident, and specialist physicians and more male physicians among consultants.

**Table 1.** Distribution of Physicians in the HealthPhys Cohort Across Hierarchical Position and Share of Women in Each Hierarchical Position.

Hierarchical position	Frequency (n)	Share of cohort (%)	Share of women (%)
Junior physician	75	2.8	57.3
Resident physician	743	27.5	61.5
Specialist physician	1080	39.9	57.3
Consultant	808	29.9	47.7

The distribution of physicians across medical specialities in the HealthPhys cohort is shown in Table 2. Those with no specialty are junior physicians who have not yet started their residency. The groups of specialties presented in Table 2 are defined according to the national recommendation by The National Board of Health and Welfare. In the last column of Table 2 the share of female physicians is presented for each specialty group. Paediatrics have the largest share of women and neurology the smallest.

**Table 2.** Number and Share of Physicians per Group of Specialty^a^.

Medical speciality	Frequency (n)	Share of cohort (%)	Share of women in the specialty
Paediatric	146	5.7	66.4%
Imaging and functional medicine	106	4.2	52.8%
Separate specialties^b^	1177	46.1	57.4%
Medicine	312	12.2	53.2%
Surgery and orthopaedics	591	23.1	48.4%
Laboratory medicine	51	2.0	58.8%
Neurology	55	2.2	34.6%
Psychiatry	115	4.5	55.7%
No specialty	75	2.9	57.3%
Missing	133	4.8	-
Total	2761	100.0	55.2%

^a^ The groups of specialties presented are those of The National Board of Health and Welfare.

^b^ Including eg, general practitioners, infectious diseases, oncology, rheumatology, emergency, occupational medicine.

In the cohort, 65.1% had a full-time contract. However, of those working full time, 7.6% used regular compensational leave to reduce weekly work hours. A larger share of primary care based than hospital-based physicians reported that they worked part time, 52.3% and 19.3% respectively. This is reflected in estimated workhours per week. Among primary care based physicians 56.9% worked 40 hours or less per week while the corresponding figure for hospital-based physicians was 24%. 39.2% of primary care based physicians worked between 41 and 50 hours per week and 3.9% more than 50 hours. The share of hospital-based physicians working between 41 to 50 hours was 61.7% and 13.7% worked more than 50 hours per week.

More than half of all physicians, 60.8% (primary care 64.1% and hospital 57.9%) stated that they need to stay after working hours daily or a couple of days a week because they had no time to finish their work during regular hours. Physicians working in primary care facilities worked part-time to a larger extent.

More than half (63.5%) of physicians in the cohort had on-call duty of which 57.6% were present at their workplace during on-call duty. When physicians are on-call they receive compensational leave and as many as 82.6% of those with on-call work, had compensational leave that they had not used. Mean hours of compensational leave saved were 196 hours (Standard deviation: 182 hours).

## What Is the Prevalence of Clinical Burnout and Major Depression among Swedish Physicians?

### Methods

#### Measurements

In this study the prevalence of clinical burnout and major depression was assessed by two scales. Major depression was assessed through SCL-CD_6_ and clinical burnout through BAT. The SCL-CD_6_ has shown good psychometric properties and is suitable for assessments of major depression in surveys.^19^ The SCL-CD_6_ is an additive scale that asks respondents about six symptoms of depressions ie, how much, during the last seven days, they have been bothered with: feeling blue/sad, no interest in things, low in energy, everything an effort, worrying too much, and blaming yourself. Answers ranged from 0 = Not at all, to 4 = Extremely. The six items are added to one variable ranging from 0 to 24. Internal consistency for SCL-CD_6_ in this study was high, Cronbach's alpha = 0.914. Following the recommendations of Magnusson Hansson et al.,^19^ cut of was set at 17 points, which is considered a suitable threshold value for major depression in epidemiological research.

BAT comprise 23 items divided into four core dimensions: exhaustion (8 items), mental distance (5 items), emotional impairment (5 items), cognitive impairment (5 items).^3^ Each item is rated on a five-point Likert scale ranging from never^1^ to always.^5^ An additive mean value was obtained for items in each core dimension. In Table 3 core dimensions are presented with internal consistency and cut-off value for a high risk of burnout as assessed by Schaufeli et al. and de Beer et al.^3,21^ The four core dimension of BAT represents symptoms of burnout and a number above the cut-off is assessed as having a very high risk of future burnout.^21^ To assess the overall degree of clinical burnout the total BAT score is used and values above 3.02 is considered to equate clinical burnout.^21^

**Table 3.** Number of Items, Cronbach's Alpha, and Cut-off for Each Dimension of BAT as Well as Total BAT.

Symptom	Number of items	Cronbach's alpha	Cut-off
Exhaustion	8	0.927	3.31
Mental distance	5	0.854	3.10
Emotional impairment	5	0.909	2.90
Cognitive impairment	5	0.857	3.10
Total BAT	23	0.862	3.02

*Gender* was divided into men and women. Age was grouped based on quartiles: 27 to 38 years, 39 to 45 years, 46 to 57 years and 58 to 77 years. *Hierarchical position* defines the hierarchical position and is divided into junior physicians, resident physicians, specialists, and consultants with consultants as the referent group. The variable *worksite* consists of hospital-based and primary care based physicians and are based on a question asking respondents whether they worked in primary care facilities or in hospitals.

#### Statistical analysis

Frequencies are used to present the characteristics of the sample and to identify the prevalence of major depression, symptoms of burnout as well as clinical burnout. When prevalence was calculated, weights were applied.

### Results

Analysis show that the prevalence of major depression among physicians in Sweden was 4.8%. Table 4 show the prevalence of major depression across specialty, hierarchical position, gender, and location of work. Across these sub-groups of physicians, the prevalence of major depression ranges from 0% to 10.1%. Results show that the prevalence of major depression was higher among female physicians (5.9%) than among male (3.7%). Junior physicians had a higher prevalence of major depression (10.1%) than more senior physicians. Across groups of specialties, Table 4 show that physicians in the psychiatric field had a 9.9% prevalence. In contrast, anaesthesiologists had a 1.8% prevalence and emergency physicians 0%.

**Table 4.** Prevalence of Major Depression, Symptoms of Burnout and Clinical Burnout Across Physicians in Sweden.

	n	Major depression (%)	Exhaustion	Mental distance	Emotional impairment	Cognitive impairment	Clinical Burnout
Gender							
Men	16 844	3.7	10.6	5.8	1.6	5.2	3.8
Women	17 530	5.9	16.5	7.1	3.0	8.1	5.5
Age groups							
27-38	9637	6.7	15.5	10.4	2.5	8.2	7.9
39-45	7565	5.9	14.3	4.5	2.1	9.4	4.1
46-57	8885	5.7	18.1	7.7	4.2	7.0	4.8
58-77	8288	0.9	5.7	2.4	0.4	2.1	1.3
Hierarchical position							
Junior physician	1027	10.1	20.5	7.3	3.4	14.2	13.0
Resident physician	8583	7.5	16.8	10.5	2.6	8.4	6.8
Specialist	10 572	4.6	15.7	6.2	1.9	8.0	4.5
Consultant	13 942	3.2	9.8	4.3	2.4	4.3	3.0
Worksite							
Primary care based	6279	3.6	16.7	8.2	1.7	7.5	5.8
Hospital-based	21 596	5.7	14.0	6.1	2.6	6.5	4.6
Selection of specialties							
Family medicine^a^	6558	4.3	14.3	7.8	2.3	7.3	5.5
Emergency	614	0	28.3	21.0	0	16.4	14.5
Anaesthesiology and intensive care	2384	1.8	5.6	2.0	2.7	2.7	1.3
Oncology	744	7.5	16.8	2.2	2.2	5.2	2.2
Medicine	4992	5.2	15.4	7.1	4.1	6.8	5.1
Surgery	1358	3.6	16.7	13.1	2.6	7.1	5.7
Orthopaedic	1691	7.6	13.4	7.6	4.4	4.3	4.7
Psychiatry	1925	9.9	22.5	9.8	1.6	14.8	10.5
Paediatric	2206	3.0	7.6	2.4	2.3	3.1	3.0
Laboratory medicine and image technology	2831	7.8	11.9	4.1	0	8.6	2.8
Physicians in Sweden (All)	34 374	4.8	13.6	6.5	2.3	6.7	4.7

^a^ Or general medicine.

The Prevalence of clinical burnout and symptoms of burnout overlap to some degree to the prevalence of major depression. Among physicians who report clinical burnout, 49,5% also report major depression (Chi^2^ test gave Pearson's R = 0.463, *P* < .001). The prevalence of clinical burnout varies across sub-groups of physicians from 1.3% to 14.5%. The prevalence of each of four dimensions representing symptoms of burnout, ie, exhaustion, mental distance, emotional impairment, cognitive impairment is presented in Table 4. In the full population of physicians, the prevalence of exhaustion is highest (13.6%). Estimates for exhaustion show that during the third wave of the pandemic many physicians experience exhaustion with prevalences ranges from 5.6% to 28.3%. Emergency physicians had the highest prevalence of exhaustion. The prevalence of mental distance and cognitive impairment was about 6% and emotional impairment 2.3%. The prevalence of exhaustion, mental distance, emotional impairment, cognitive impairment also varies to a large extent across sub-groups of physicians. Some estimates are worth highlighting. For instance, resident physicians have higher prevalence of mental distance that junior physicians (21%) while no emergency physician present symptoms of emotional impairment (0%).

## Discussion

The HealtPhys study provide data that is representative of Swedish physicians and has the potential to make important contributions to the field of research both nationally and internationally.

The present study shows the prevalence of clinical burnout and major depression among physicians in Sweden and across sub-groups of physicians which adds valuable knowledge to previous research. The Prevalence of major depression among physicians in Sweden in relation to the general Swedish working population seem to be somewhat lower. Swedish studies applying the SCL-CD_6_ instrument to assess major depression show a prevalence of 8.5% in the general population in Stockholm.^19^ Meanwhile, Wurm et al.^22^ present a 7.2% prevalence of major depression among physicians in Austria.

The results for major depression, clinical burnout as well as symptoms of burnout (exhaustion, mental distance, emotional impairment and cognitive impairment) show variations across gender, age, hierarchical position, worksite, and specialty which are in line with previous studies.^1,22^ Among the four dimensions on clinical burnout, estimates on exhaustion was overall highest. Exhaustion is one primary symptom of clinical burnout and signifies extreme tiredness, and severe and serious loss of energy, both physical as well as mental.^3^ The high prevalence of exhaustion among emergency physicians could be a result of the high workload during the pandemic, as indicated in previous studies.^23^ In future studies, we will explore these differences in more details.

Previous studies show that depression and burnout co-occur and develop in tandem.^22,24^ In the present study, statistics indicate an overlap between major depression and clinical burnout but to a lesser degree than among physicians in Austria.^22^ Although this need further evidence, we hypothesis that in this population of physicians, clinical burnout or rather exhaustion was an effect of the high demands during the Covid-19 pandemic. In future studies we will explore antecedents to prevalence of major depression and clinical burnout among practising physicians in Sweden in cross-sectional and longitudinal studies with the over-all purpose to reduce occupational ill health.

For employers, it is important to highlight the high prevalence of major depression and clinical burnout among junior physicians as well as among those working within psychiatry. Junior physicians are the future attending physicians and consultants. To be able to work their whole life, measures need to be taken.

This study was conducted during spring 2021, at the peak of the third wave of the pandemic and results should be considered in the light of this. For instance, the HealthPhys study show that many physicians experienced high levels of exhaustion a year after the start of the pandemic indicating that they are at high risk of burnout. This is the first study in Sweden capturing work and health among front-line healthcare workers. Based on the HealthPhys study, we will in future studies be able to draw conclusions of the work conditions of physicians in Sweden during the Covid-19 pandemic, and the possible health effects thereof. Moreover, we will explore variations across specialties, regions, and healthcare facilities. In relation to the Covid-19 pandemic, in a future study, the occupational exposures and health related to the pandemic in specific will be further explored.

### Methodological Discussion

There exist various validated instruments that measure various aspects of mental health. In the Swedish HealthPhys study, we applied BAT to assess clinical burnout and SCL-CD_6_ to assess major depression. BAT was developed by Schaufeli et al.^3^ in response to identified limitation in the often-used Maslach Burnout Inventory (MBI). Schaufeli and colleagues argue that MBI have problems with the conceptualisation of burnout, psychometric shortcomings, and that the practical applicability for individual burnout is poor especially in a work context. A reason for way BAT was applied in the HealthPhys project is that the composite score indicate clinical burnout whereas MBI do not produce one single burnout score.^3^ Most international articles studying the prevalence of clinical burnout among practising physicians have applied MBI. However, concerns of the validity of MBI have been raised in response to the vide range in the prevalence of burnout among physicians based on MBI measurements.^1^ Furthermore, Wurm et al.^22^ argue that research should not rely on MBI exclusively to assess burnout. The main reason that SCL-CD_6_ was used instead of eg, Patient Health Questionnaire (PHQ-9) is that in the pilot of the questionnaire, physicians expressed a discomfort in using the PHQ-9 as it is an instrument used to diagnose their patients. Furthermore, SCL-CD_6_ was chosen to assess major depression as it is applied in the Swedish Longitudinal Occupational Survey of Health (SLOSH) as well as in the Norwegian study on the medical professions. This allows for comparisons between physicians in Sweden and the Swedish working population (SLOSH) as well as the Norwegian physicians. A major strength of the SCL-CD_6_ is that it has high clinical and construct validity and is brief in relation to other depression scales.^19^

This study is the first data collection, and a follow-up survey will be collected on individual-level in March of 2022 creating a longitudinal cohort. As such, the HealthPhys study will be able to follow trends over time as well as the effect of the Covid-19 pandemic on physicians working conditions and health. Furthermore, the longitudinal HealthPhys database will be able to identify occupational risk factors that causes poor health. Rotenstein et al.^1^ conclude that there is a need for such longitudinal datasets that capture the broader adverse effects of physician stress, depression, anxiety, and substance abuse along with consistent measures of occupational factors that shape the physicians working life. Finally, the HealthPhys study will also be linked to registered base sick leave data from registers held by the Swedish Social Insurance Agency. Alone, the HealthPhys study will be able to identify risk factors for sickness absence.

## Conclusions

The HealthPhys study includes a representative sample of physicians working in Sweden. As such, it is a unique cohort in Sweden and, as far as we can see, also in an international perspective except for Norway. The Prevalence of both major depression and clinical burnout in practising physicians in Sweden varies across, gender, age, worksite, hierarchical position, and specialty. Data from the HealthPhys study indicate that many physicians are exhausted and at risk of clinical burnout and that measures urgently needs to be taken to reduce such risks. The HealthPhys study will make valuable contributions to the field of research and even more so when the second wave of data is collected in spring 2022. 
